# Metabolomic and Molecular Mechanisms of Glycerol Supplementation in Regulating the Reproductive Function of Kazakh Ewes in the Non-Breeding Season

**DOI:** 10.3390/ani15152291

**Published:** 2025-08-05

**Authors:** Ying Nan, Baihui Jiang, Xingdong Qi, Cuifang Ye, Mengting Xie, Zongsheng Zhao

**Affiliations:** College of Animal Science and Technology, Shihezi University, Shihezi 832000, China; 17699532990@163.com (Y.N.); m18899530621@163.com (B.J.); q15117259643@163.com (X.Q.); yeshz@shzu.edu.cn (C.Y.); 17699732156@163.com (M.X.)

**Keywords:** estrus induction, glycerol, lipid metabolism reprogramming, kisspeptin/GnRH pulse, metabolic–neural axis regulation

## Abstract

The activation mechanism of the reproductive axis in Kazakh ewes during the non-breeding season was explored by supplementation with glycerol complex (7% glycerol + tyrosine + vitamin B9). The experiment divided 50 ewes into five groups (*n* = 10). After 90 days of intervention, it was found that significant changes in serum DL-carnitine, N-methyl-lysine and other differential metabolites were observed in the GLY-Tyr-B9 group (*p* < 0.05, “*p* < 0.05” means significant difference, “*p* < 0.01” means “highly significant difference”). The bile acid metabolic pathway was specifically activated (*p* < 0.01). The group had a 50% estrus rate, ovaries contained 3–5 immature follicles, and HE staining showed intact granulosa cell structure. Serum E2/P4 fluctuated cyclically (*p* < 0.01), FSH/LH pulse frequency increased (*p* < 0.01), peak Glu/INS appeared on day 60 (*p* < 0.05), and LEP was negatively correlated with body fat percentage (*p* < 0.01). Molecular mechanisms revealed: upregulation of hypothalamic kiss-1/GPR54 expression (*p* < 0.01) drove GnRH pulses; ovarian CYP11A1/LHR/VEGF synergistically promoted follicular development (*p* < 0.05); the HSL of subcutaneous fat was significantly increased (*p* < 0.05), suggesting involvement of lipolytic supply. Glycerol activates the reproductive axis through a dual pathway—L-carnitine-mediated elevation of mitochondrial β-oxidation efficacy synergizes with kisspeptin/GPR54 signalling enhancement to re-establish HPO axis rhythms. This study reveals the central role of metabolic reprogramming in regulating seasonal reproduction in ruminants.

## Simple Summary

This study focuses on seasonal reproduction in sheep, the core problem of efficient production, and innovatively proposes a theoretical framework of “metabolic reprogramming for estrus”. Because the key metabolic nodes and interactions across tissues in nutrition-induced estrus in non-breeding ewes have not been elucidated, this project uses glycerol as a metabolic intervention carrier and reveals three major scientific points through the strategy of metabolomics integration: (1) How energy signalling regulates the activation of Kisspeptin/GPR54 signalling in the hypothalamus through metabolite-hormone axes; (2) The lipolytic metabolite (L-carnitine) cooperates with the microenvironment of ovarian steroid production to promote follicular development; (3) The contribution of the bile acid–metabolism axis to seasonal reproductive regulation. The research results will be the first to map out the “metabolite–neuroendocrine–reproductive phenotype” panoramic regulatory network, providing an innovative theoretical paradigm for the precise nutritional control of the reproduction cycle in ruminants.

## 1. Introduction

Seasonal reproduction is an important physiological feature for ruminants to adapt to the environment. However, its resulting reproductive inefficiency has become a key bottleneck restricting sheep industry development. Studies have shown that ewes are generally in a state of negative energy balance (Body Condition Score BCS < 2.5) during the spring to early summer period (March–June), which triggers the inhibition of the hypothalamic Kisspeptin/GnRH signalling pathway, ultimately leading to functional stagnation of the HPG axis [1]. This phenomenon is closely associated with impaired follicular development: in the energy-deprived state, sinus follicle diameter is reduced by 40% and granulosa cell apoptosis is elevated to 68%, while follicular fluid IGFBP-3 concentration is significantly increased (3.2 ± 0.4 vs. 1.8 ± 0.3 ng/mL), inhibiting the follicle maturation-promoting effect of IGF-1 [2].

Nutritional interventions are an effective strategy to overcome seasonal reproductive constraints, focusing on re-establishing reproductive endocrine homeostasis through metabolic reprogramming [3,4,5]. Notably, energy-rich feeds (e.g., glycerol) demonstrate significantly higher proestrus induction efficiency (50–70%) than protein-rich feeds (20–30%), indicating a pivotal role of energy metabolism pathways in reproductive regulation [6]. Previous studies demonstrated that a 7-day oral glycerol intervention significantly increased the ovulation rate in Manchega ewes, primarily via enhanced β-oxidation-derived energy supply and elevated follicular fluid IGF-1 concentrations [7]. This finding suggests that energy metabolism reprogramming regulates seasonal reproductive rhythms through the TH-DIO2/DIO3-GnRH and Kisspeptin/GnRH dual signalling systems. Negative energy homeostasis in females directly inhibited Kisspeptin neuron activity in the arcuate nucleus of the hypothalamus, leading to a decrease in the frequency of GnRH pulses. Meza-Herrera’s team further demonstrated that short-term ad libitum feeding (metabolizable energy intake ↑ 30%) promotes corpus luteum function and coordinates LH pulse secretion (amplitude ↑ 40%, *p* < 0.01) [8]. Leptin acts as a metabolic–reproductive signalling hub, and Leptin regulates energy balance in both directions through the JAK2/STAT3 pathway. Exogenous leptin (5 mg/kg BW) advanced the onset of GnRH pulses in preprimary rats by 3.2 days (*p* < 0.01), and energy restriction led to a decrease in adipose tissue Leptin receptor (LEPR) expression by 52%, triggering leptin resistance [9]. Notably, Leptin can directly act on hypothalamic POMC neurons through the blood-brain barrier, activating the ERK1/2 signalling cascade (p-ERK ↑ 3.5-fold) to promote GnRH release [10]. Collectively, these findings demonstrate that leptin coordinates metabolic–reproductive homeostasis by modulating hypothalamic–pituitary–gonadal (HPG) axis function, establishing a mechanistic basis for targeted nutritional interventions.

In recent years, significant breakthroughs have been achieved in understanding seasonal reproduction regulation mechanisms. Yang et al. [11] first revealed the central regulatory role of the GNAQ gene in the hypothalamic–pituitary–ovarian (HPG) axis by constructing a multi-omics database (miRNA/mRNA) of estrus and anestrus in Kazakh sheep during the non-breeding season. This gene is highly expressed across all three levels of the reproductive axis and functions through bidirectional regulation: ① Regulating key enzymes in vitamin B9 metabolism (MTHFR for methyltetrahydrofolate reductase) and tyrosine hydroxylase activity to remodel reproductive hormone secretion rhythms; ② Exhibiting a highly significant positive correlation with light signal intensity (*p* < 0.01), indicating its role as a “light-nutrient integration node”. Building on this regulatory framework, Lin et al. [12] systematically analyzed molecular events in the HPG axis using a high-nutrient diet-induced estrus ewe model. Their study demonstrated that Tyr and B9 synergistically induce estrus via the leptin–estrogen axis independently of photoperiod, with spatiotemporal-specific mechanisms—Epigenetic regulation: B9 promotes ovarian steroid synthesis through DNMT3B-mediated DNA demethylation (phosphorylation sequencing revealed 40% reduction in CYP19A1 promoter methylation) [13]; Cellular signaling: Tyr drives granulosa cell proliferation and LH receptor expression by activating the NTRK2/PI3K-AKT pathway [14,15]. These findings collectively support a “nutrient-epigenetic crosstalk” model for seasonal reproduction regulation, providing a novel theoretical framework for ruminant reproductive management. This mechanism was validated in a cross-species study: an L-tyrosine supplementation intervention (1.0 g/kg BW) was administered to ewes 7 days prior to the initiation of the breeding season, and it was found that the estrous response rate of ewes in the experimental group was significantly higher than that of the control group [16] and that the B9 intervention in gestating ewes led to an increase in the number of live born piglets in the litter by 2.3 (*p* < 0.01) [17].

In this study, we focused on the metabolomic differences between GLY and traditional nutritional regulatory mechanisms: (1) using LC-MS to screen for serum differential metabolites, focusing on bile acids and lysophospholipids metabolic profiles, and (2) constructing a three-level regulatory network of “metabolite-methylation modification-hormone pulse”. This study reveals the central role of metabolic reprogramming in regulating seasonal reproduction in ruminants.

## 2. Materials and Methods

### 2.1. Animals, Experimental Design, and Diets

In this study, 50 healthy parturient Kazakh ewes (3–5 years old, weighing 38.0 ± 0.92 kg) were selected and divided into five groups (*n* = 10/group) using a completely randomized design: Control group (basal diet, NRC 2007 standard [18], Table 1), GLY group (basal diet + 7% GLY (Dry matter basis, converted to 70 mL/head/day), purity ≥ 99.5%), GLY-Tyr group (basal diet + 7% GLY + 100 mg/kg Tyr, Sigma-Aldrich^®^ (Saint Louis, MO, USA) CAS 60-18-4), GLY-B9 group (basal diet + 7% GLY + 6 mg/kg B9, Sigma-Aldrich^®^ CAS 59-30-3) and GLY-Tyr-B9 group (basal diet + 7% GLY + 100 mg/kg Tyr + 6 mg/kg B9). During the non-breeding season (1 March–30 May 2023), a daily gavage intervention was administered at 10:00 a.m. for 90 consecutive days.

Before the test, the pen was thoroughly cleaned and disinfected, and the sheep were grouped by vaccination and deworming status, as well as by shearing and weighing. They were then kept in semi-open sheds with consistent environmental conditions (temperature, ventilation, and light).

### 2.2. Sample Preparation and Analyses

In this study, we used a multi-dimensional method to assess the estrus status of ewes systematically, including (1) ligated ram estrus test method (daily 07:00/20:00, recording the acceptance of crawling across the behavior); (2) vulvar behavioral observation; (3) reproductive hormone test (E2, P4, LH, and FSH; the kits were purchased from Jiangsu Jingmei Biotechnology Co., Ltd., Yancheng, China): (4) Nanjing Jianzhong Bioengineering Institute, Nanjing, China provided Glu, INS, LEP detection kits. Samples (5× diluted) and standards were incubated with enzyme conjugate (37 °C, 60 min), washed five times, developed with TMB substrate (15 min), stopped, and read at 450 nm. Concentrations were calculated against standard curves.

At the end of the experimental cycle, the hypothalamus, ovary, and subcutaneous adipose tissues were collected from estrous ewes of the test group and non-estrous ewes of the control group, then passed through the Total RNA (A260/A280 = 1.8–2.0) was extracted using the Tissue RNA Extraction Kit (Thermo Fisher Scientific, Beijing, China). cDNA was synthesized by HiFiScript gDNA Removal RT MasterMix (CWBIO, Taizhou, China). The qRT-PCR reaction system was 25 μL (containing 12 μL of SYBR Premix EX Taq II, 1 μL each of forward/reverse primers, 2 μL of cDNA template, 9 μL of RNase-Free ddH_2_O), and the amplification program was set as follows: pre-denaturation at 95 °C for 5 min; 40 cycles of 95 °C 10 s denaturation → 60 °C 30 s annealing/extension; and the amplification specificity was determined by the unstranding temperature (Tm value) ± 0.8 °C at the melting curve analysis stage. A dual-platform validation using ABI PRISM 7500 (Applied Biosystems, Waltham, MA, USA) and Roche LightCycler480 II (Roche Diagnostics, Rotkreuz, Switzerland) was used to standardize the assay using sheep β-actin as an internal reference gene and to detect reproduction-related genes (GnRH, GnAQ, kiss-1, GPR54, CYP11A1, LHR, VEGF, and StAR). Lipid metabolism genes (HSL) were differentially expressed (primer sequences are shown in Table 2, validated by NCBI Primer-BLAST (https://blast.ncbi.nlm.nih.gov/Blast.cgi, accessed on 15 February 2024)).

### 2.3. Serum Non-Targeted Metabolomics

On the 90th day of the test, 5 mL of jugular vein blood was obtained from estrous ewes in the test group and non-estrous ewes in the control group (*n* = 6). Serum was separated and submitted to Novozymes Biotechnology Co., Ltd., (Shanghai, China) for UHPLC-QTOF-MS analysis. Aliquots of 100 μL serum were mixed with 400 μL of 80% (*v*/*v*) aqueous methanol, followed by vortexing and centrifugation at 15,000× *g* for 20 min at 4 °C. The supernatant was diluted to 53% (*v*/*v*) methanol content and recentrifuged under identical conditions.

Chromatographic separation was employed with a Waters ACQUITY UPLC system equipped with a Hypersil Gold C18 column (100 × 2.1 mm, 1.9 μm; Thermo Fisher) using 0.1% (*v*/*v*) formic acid in water (mobile phase A) and acetonitrile (mobile phase B) with a 16 min gradient at 0.2 mL/min. Mass spectrometric detection was performed on a Q Exactive HF-X mass spectrometer (Thermo Fisher) with electrospray ionization (spray voltage: 3.5 kV). Full scans (*m*/*z* 100–1500) were acquired in both positive and negative modes from 0–12 min with the following parameters: auxiliary gas flow 10 L/min, capillary temperature 320 °C, S-lens RF level 60, heater temperature 350 °C, and data-dependent MS/MS acquisition.

### 2.4. Statistical Analyses

Metabolomics data were analyzed using the following process: raw mass spectrometry data were pre-processed by Compound Discoverer 3.1 software, and the characteristic peaks were screened by retention time deviation (±0.2 min) and mass-to-charge ratio deviation (≤5 ppm) and combined with the molecular peaks and fragmentation ions to match the mzCloud, mzVault and Masslist databases. Metabolite characterization was completed using KEGG (https://www.genome.jp/kegg/pathway.html, accessed on 15 February 2024), HMDB (https://hmdb.ca/metabolites, accessed on 15 February 2024), and LIPIDMAPS (http://www.lipidmaps.org/, accessed on 15 February 2024) databases for metabolic pathway annotation, and reliable quantitative results were obtained by QC sample quality control (CV < 30%). After data standardization (relative peak area method) using metaX software 2.33, multivariate statistical analyses were performed: principal component analysis (PCA) to reveal the trend of separation between groups, partial least squares-discriminant analysis (PLS-DA) to calculate the projected importance of the variables (VIP value), and the combination of univariate *t*-tests (*p* < 0.05) with the multiplicity of variance (FC > 1.5 or <0.8) to screen for significantly different Metabolites. Statistical analyses were performed using R 4.3 (ggplot 2 package to plot volcano/bubble plots; complot package 1.8 to display Pearson correlation matrices) and Python 3.10 (scikit-learn machine learning library); the significance of difference validation was performed by Duncan multiple comparisons (*p* < 0.05) using SPSS 26.0, and visualization was performed using GraphPad Prism 9.0.

qRT-PCR data were processed by the 2^−ΔΔCt^ method, and one-way analysis of variance (ANOVA) with Duncan’s multiple comparisons (SPSS 26.0) was used, and the results were expressed as mean ± standard error (“*p* < 0.05” indicates significant difference, which is labeled with “*” in the figure, and “*p* < 0.01” indicates “highly significant difference, which is labeled with “**” than labeled), and the final graphs were completed by GraphPad Prism 9 Drawing. Three biological replicates were set for each treatment, and three technical replicates were performed for each biological replicate.

## 3. Results

### 3.1. Effect of Glycerol Mixtures on Estrus Rate of Ewes in the Non-Breeding Season

**Figure 1:**
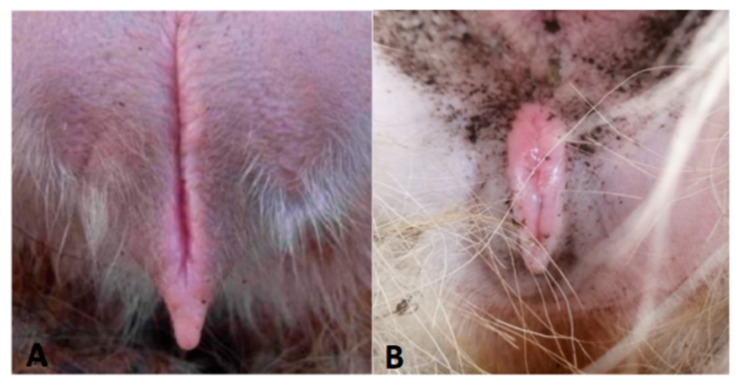
Vulva of a spent ewe (**A**), vulva of an estrous ewe (**B**).

**Figure 2:**
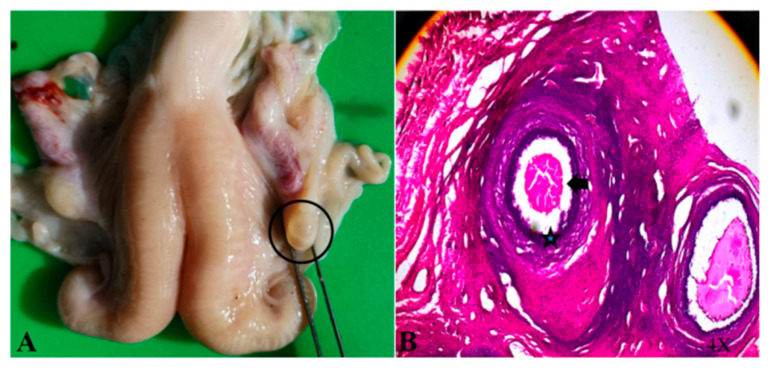
Ovary of an estrous ewe (**A**) and HE-stained section (**B**).

In the non-breeding season, ewes supplemented with glycerin mixture all show the initial behavior (approaching the ram, wagging their tails, and riding to accept), and their vulva is red and secretes mucus (Figure 1). The GLY-Tyr-B9 group showed optimal estrus induction (50% estrus rate, Table 3). Dynamic hormone monitoring revealed pulsatile E2 fluctuations synchronized with LH/FSH secretion rhythms (*p* < 0.01), alongside fluctuating P4 levels (*p* < 0.01). Ovarian morphology indicated 3–5 immature follicles (2.3 ± 0.5 mm diameter) in estrous ewes. HE staining demonstrated structurally intact primary follicles: deeply stained oocyte nuclei (hematoxylin), eosinophilic cytoplasm (eosin), tightly arranged monolayered granulosa cells (Figure 2A, arrowheads), and characteristic hyaline bands between oocytes and granulosa cells (Figure 2B, asterisks).

### 3.2. Regulatory Effects of Glycerol Complexes on Reproductive Hormone Secretion Patterns in Ewes

**Figure 3:**
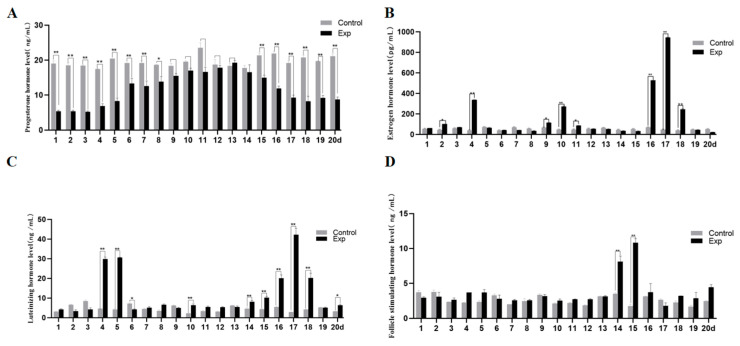
Hormone levels in the estrous cycle of ewes. (**A**) P4 hormone level, (**B**) E2 hormone level, (**C**) LH hormone level, and (**D**) FSH hormone level. Note: “*p* < 0.05” indicates a significant difference, which is labeled with “*” in the figure, and “*p* < 0.01” indicates “highly significant difference, which is labeled with “**” in the figure.

Dynamic monitoring of serum reproductive hormones revealed that non-breeding season-induced estrous ewes exhibited a simulated artificial cycle [19] (Figure 3). P4 dynamics: Progressive increase from day 4, peaking at day 13 (18.8 ± 1.2 ng), followed by decline to baseline by day 18, mimicking natural luteal phase patterns. E2 pulsatility: Significant peaks at days 4, 10, and 16–18 (*p* < 0.01), contrasting with low-fluctuating CON levels. LH/FSH synchronization: LH surges at days 4–5, 10, 14–18, and 20 (*p* < 0.01); FSH peaks at days 14–15 (*p* < 0.01), phase-synchronized with LH. Glycerol complex induces estrus of ewes in the non-breeding season through the HPO axis, achieving a 50% estrus rate in the GLY-Tyr-B9 group.

### 3.3. Dynamic Regulation of Lipid Metabolism Hormones in Ewes by Glycerol Complex

**Figure 4:**
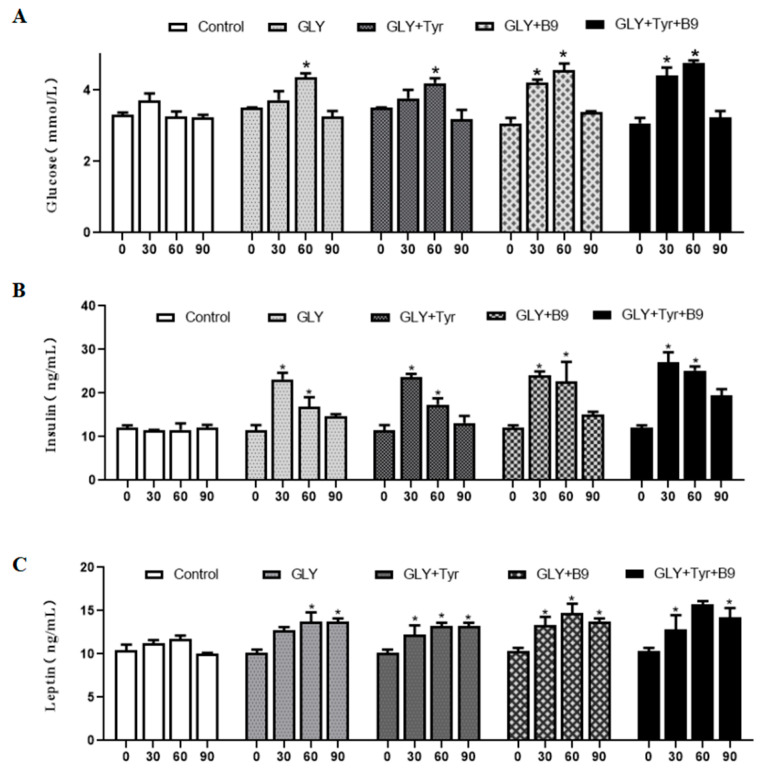
Hormonal changes in lipid metabolism. (**A**) Glu levels. (**B**) INS levels. (**C**) LEP levels. “*p* < 0.05” indicates a significant difference, which is labeled with “*” in the figure.

At the beginning of the experiment (day 0), no significant differences in glucose (Glu), insulin (INS), or leptin (LEP) levels were observed among groups (*p* > 0.05). Dynamic monitoring revealed (Figure 4). Glu: All groups exhibited an initial increase followed by decline (*p* < 0.05); INS: GLY, GLY-Tyr, and GLY-B9 groups peaked at day 30 (22.7 ± 1.5 ng/mL, *p* < 0.05), maintaining higher levels vs. controls at day 60 (16.4 ± 2.1 ng/mL, *p* < 0.05) despite a decrease from peak. The GLY-Tyr-B9 group showed a 28.7% higher INS peak vs. GLY alone (*p* < 0.05); LEP: Progressive rise to 14.2 ± 0.5 ng/mL at day 60 (*p* < 0.05) after transient elevation (13.8 ± 0.3 ng/mL at day 30, *p* > 0.05), stabilizing at 13.5±0.4 ng/mL by day 90. These findings indicate that Glu, INS, and LEP dynamics correlate with energy intake and are modulated by synergistic nutrient interactions, driving adipose accumulation and leptin feedback enhancement.

### 3.4. Regulatory Effects of Nutritional Interventions on Gene Expression in the Reproductive-Metabolic Axis of Sheep

**Figure 5:**
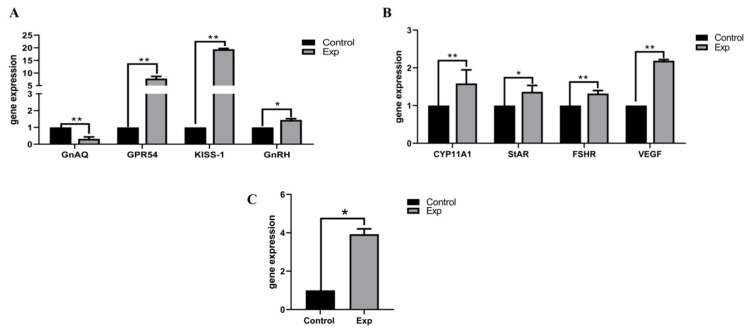
Gene expression quantity. (**A**) Oestrus gene expression in hypothalamic tissue; (**B**) Oestrus gene expression in ovarian tissue; (**C**) Oestrus gene expression in subcutaneous adipose tissue. “*p* < 0.05” indicates a significant difference, which is labeled with “*” in the figure, and “*p* < 0.01” indicates “highly significant difference, which is labeled with “**” in the figure.

qRT-PCR analysis revealed supplementary feeding-induced regulatory effects on reproductive-metabolic genes in non-breeding season ewes (Figure 5): Hypothalamic pathway: GNAQ was significantly downregulated (*p* < 0.01), suggesting its role as a photoperiod-sensitive node in estrus suppression. The kisspeptin system exhibited cascade activation: Kiss1 ↑ (*p* < 0.01) → GPR54 ↑ (*p* < 0.05) → GnRH ↑ (*p* < 0.05), establishing a “GNAQ-Kiss1-GnRH” regulatory axis. Ovarian steroidogenesis: Upregulation of StAR (*p* < 0.05) and CYP11A1 (*p* < 0.01) accelerated cholesterol-to-pregnenolone conversion. Follicular development: LHR and VEGF upregulation (*p* < 0.01) enhanced angiogenesis and luteal functionality. Lipid mobilization: HSL upregulation (*p* < 0.05) redirected energy metabolism toward reproductive allocation.

### 3.5. Metabolomics QC and PCA

**Figure 6:**
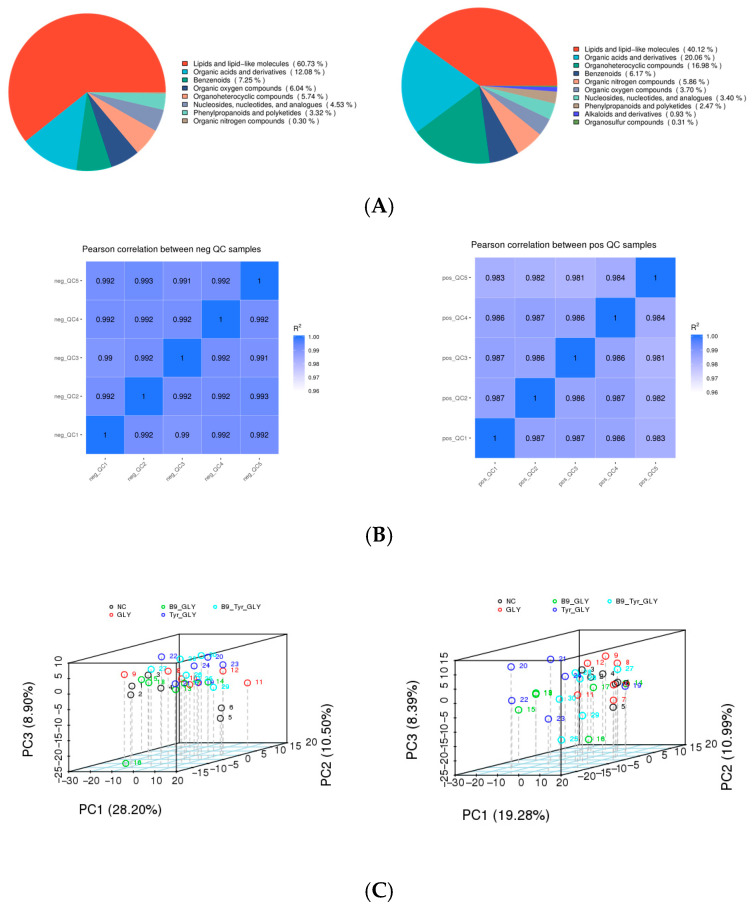
(**A**) Metabolite classification. (**B**) QC sample correlation analysis. (**C**) Total sample PCA analysis.

Untargeted serum metabolomics using UHPLC-QTOF-MS identified 1082 metabolites (ESI+: 618; ESI−: 464). Lipid and lipid-like molecules dominated the metabolic profile (60.7%), followed by amino acids/derivatives (27.2%) and organic acids (12.1%) (Figure 6A). High reproducibility was confirmed by Pearson correlations (Figure 6B). PCA revealed significant metabolic divergence between control and experimental groups (GLY, GLY-Tyr, GLY-B9, GLY-Tyr-B9) along PC1/PC2 (*p* < 0.01), with tight intra-group clustering (>90% variance explained), demonstrating robust system stability (Figure 6C).

### 3.6. Metabolomics Multivariate Statistical Analysis

**Figure 7:**
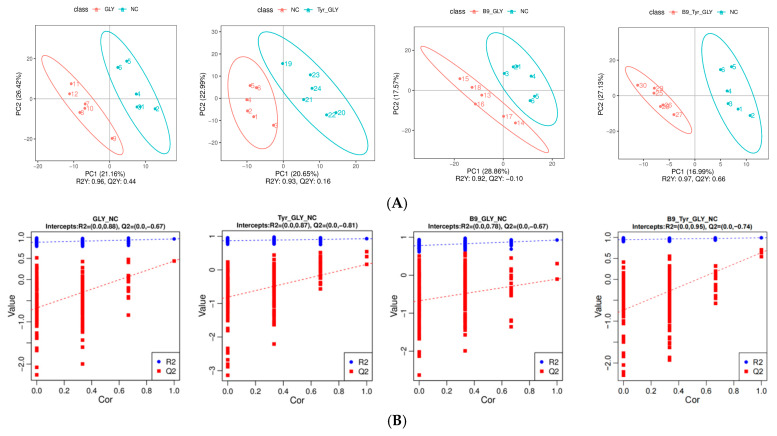
(**A**) PLS-DA Score Scatter Plot. (**B**) Ranked Validation Plot.

PLS-DA modeling revealed that supplemental feeding significantly remodeled ewe metabolic profiles. Model validation demonstrated high goodness-of-fit (R^2^ > 0.92) with predictive reliability (Q^2^ = 0.10–0.66, R^2^ > Q^2^). Permutation tests confirmed robustness (*p* < 0.001, slope < 0.3). PLS-DA score plots showed clear metabolic separation between experimental groups (GLY, GLY-Tyr, etc.) and the NC group (*p* < 0.01), with tight intra-group clustering (Hotelling T^2^ > 95%) (Figure 7), indicating glycerol complexes specifically modulate metabolic networks.

### 3.7. Differential Metabolite Screening and Functional Analysis

**Figure 8:**
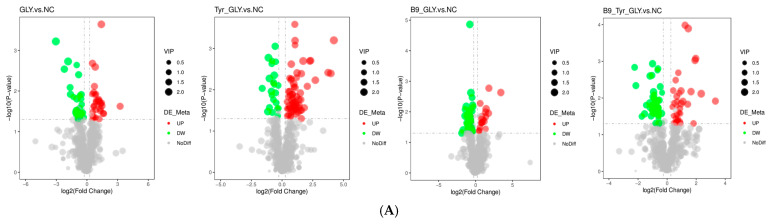

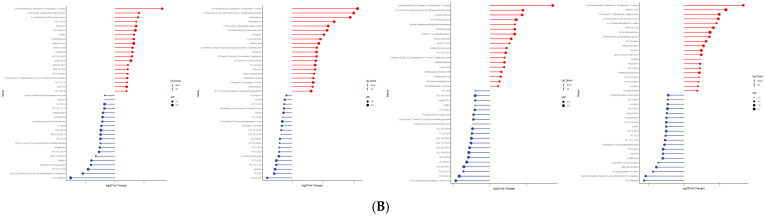
(**A**) Volcano diagram. (**B**) Matchstick diagram.

Based on LC-MS/MS non-targeted metabolomics technology, 281 differential metabolites were identified using variable importance projection (VIP > 1.0), fold difference (FC > 1.2 or FC < 0.833) and statistical significance (*p* < 0.05) as screening criteria. Visualized by volcano plot (Figure 8A) and matchstick plot (Figure 8B), it was found that: Gly vs. NC group: 30 metabolites were significantly upregulated (e.g., lysophosphatidylcholine ↑), and 27 were significantly downregulated (e.g., leucine ↓ 35%); B9-GLY vs. NC group: 18 were upregulated (ergosterol ↑), and 42 were downregulated (taurocholic acid ↓); Tyr -GLY vs. NC group: 62 upregulated (DL-carnitine ↑), 23 downregulated (glutamine ↓); B9-Tyr-GLY vs. NC group: 35 upregulated (arachidonic acid ↑), and 44 downregulated (tryptophan ↓).

**Figure 9:**
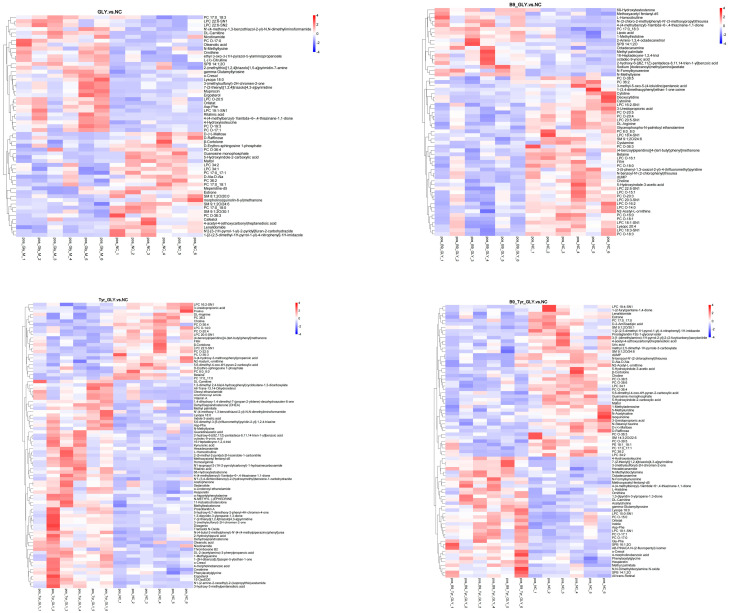
Clustering heat map of differential metabolites.

**Figure 10:**
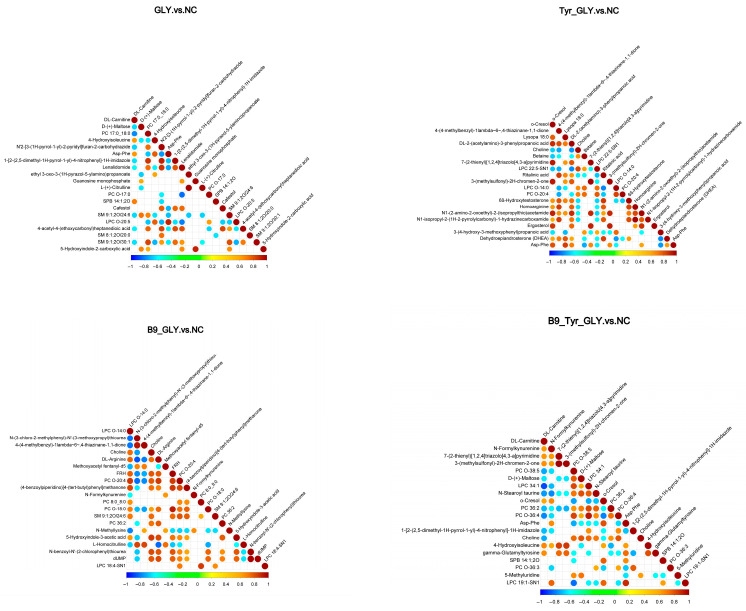
Correlation diagram of differential metabolites.

Differential metabolites were enriched in three major categories (Table 4): glycerophospholipids, fatty acids/derivatives, and amino acids/metabolites. Cluster analysis (Figure 9) and correlation analysis (Figure 10) identified DL-carnitine as a core metabolite with multifunctional roles. Energy supply: Enhanced mitochondrial β-oxidation (ATP), supporting follicular development; Ovarian activation: Stimulated IGF-1/PI3K-AKT pathway (Progesterone ↑); Methylation synergy: Promoted lysine methylation (N-methyllysine ↑) with B-vitamin cofactors (CoA ↑; B5 ↑); Biosynthesis regulation: N-methyllysine modulated lysine substrate availability, elevating DL-carnitine biosynthesis. These results demonstrate multilevel metabolic–reproductive axis regulation by nutritional interventions.

### 3.8. Functional Analysis and Regulatory Network of Metabolic Pathways

**Figure 11:**
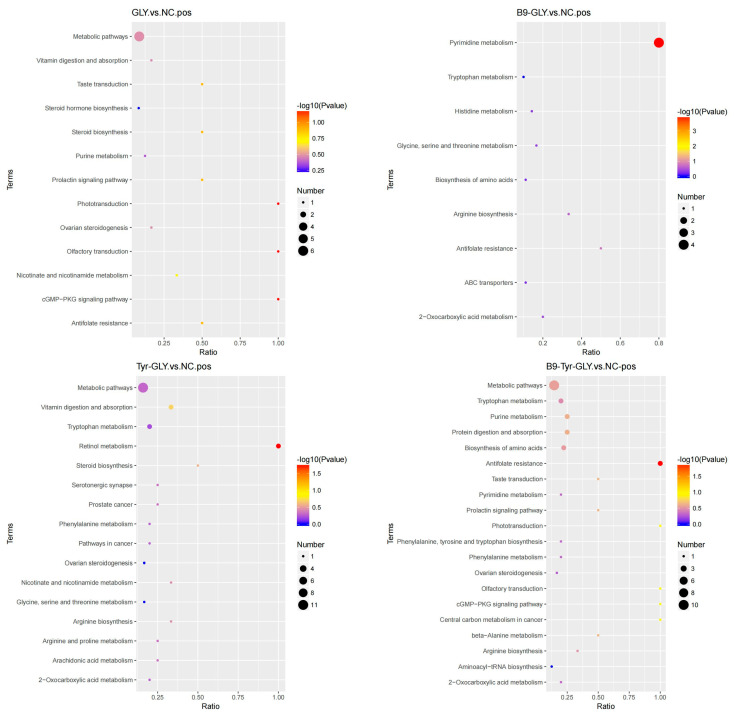
KEGG enrichment bubble plot.

KEGG pathway analysis (Table 5, Figure 11) revealed differential metabolites enriched in lipid/energy/amino acid metabolism. Gly group: Steroid biosynthesis (CYP11A1 ↑, Pregnenolone ↑) with suppressed prolactin signaling (PRL ↓) synergistically reset estrous cyclicity. Tyr-Gly group: Retinol metabolism activated RARα-mediated follicular maturation (E2 ↑). B9-Gly group: Folate metabolism reduced CYP19A1 promoter methylation while ABC transporters enhanced cholesterol flux (HDL ↑). B9-Tyr-Gly group: Multipathway synergy (DL-Carnitine ↑, VEGF ↑) promoted ovarian angiogenesis via cGMP-PKG signaling, with arginine-driven polyamine synthesis supporting proliferation. The glycerol complex induced non-seasonal estrus through three-tiered regulation: Lipid initiation: Steroid/bile acid cycles provided hormonal precursors. Energy adaptation: Retinoic/folate axes optimized follicular energy homeostasis. Epigenetic remodeling: DNMT3B-mediated CYP19A1 demethylation synergized with Kisspeptin/GnRH pulses. This establishes a “metabolism–epigenetic–neural” framework for regulating ruminant seasonal reproduction.

## 4. Discussion

### 4.1. Effectiveness of Glycerol Complex in Inducing Estrus in Ewes During the Non-Breeding Season

In the present study, all the experimental groups supplemented with glycerol complex successfully induced estrous behaviors [20], with the rate of estrus in the GLY-Tyr-B9 group being significantly higher than that of the other treatment groups (50% vs. 30–35%). Ovarian morphology showed 3–5 primary follicles (2.3 ± 0.5 mm in diameter) in the right ovary of oestrous individuals, and HE-stained sections further confirmed that the follicles were in the primary developmental stage. This phenomenon is consistent with the study of Zhu Mengting et al. [6], suggesting that high-energy supplementation can improve the ovarian microenvironment and promote early follicular development through metabolic reprogramming.

### 4.2. Remodeling Effects of Glycerol Complex on Reproductive Hormone Rhythms in Ewes

FSH and LH, as glycoprotein hormones secreted by the pituitary gland, play a central role in mammalian reproductive activities by synergistically regulating the processes of follicle recruitment, dominance and maturation [21,22]. In this study, we found that supplemental feeding of glycerol complex during the non-breeding season significantly remodelled the reproductive endocrine network in ewes: FSH in ewes of the supplemented group showed a typical gonadotropin pulsatile secretion (peak 12.5 ± 0.7 ng/mL vs. 1.8 ± 0.3 ng/mL in the CON group). Its fluctuation was significantly higher than that of the blank control group (CON group) (*p* < 0.01), which was in high agreement with the hormone during the oestrous period of the Parmesan meat goat pattern and was highly consistent [23]. Seasonal monitoring data of Kazakh ewes further confirmed that the basal level of FSH was 1.8-fold higher (*p* < 0.01) during the estrous period compared with that during the anaestrus period [24], suggesting that nutritional intervention can mimic physiological reproductive rhythms. A significant E2 peak appeared on day 17 in the supplemented group (913.65 ± 39.63 pg/mL vs. 22.7 ± 3.8 pg/mL in the CON group, *p* < 0.01), which was consistent with the evolutionarily conserved feature of 72-fold elevation of E2 levels during the reproductive period of Sichuan snub-nosed monkeys [25]. P4 levels peaked at 18.8 ± 1.2 ng/mL on day 13, with the dynamic profile mirroring luteal phase characteristics of natural estrous cycles in Haribaqing ewes [26]. These findings demonstrate the physiological recapitulation of glycerol mixture-induced estrus during non-breeding seasons. Steroid hormones suppress LH secretion by inhibiting Kisspeptin neuronal activity under nutritional deprivation [27]. In this study, glycerol-based intervention induced hyperglycemia to stimulate pancreatic β-cell insulin secretion. Promoting free fatty acid (FFA) esterification into triglycerides, thereby triggering lipid accumulation and subsequent LEP elevation [28]. This cascade re-established hypothalamic–pituitary–gonadal (HPG) axis activation (GnRH pulsatility), overcoming “metabolic–reproductive decoupling” in seasonal breeding. Our findings propose a novel nutritional strategy for off-season estrus induction in ruminants via metabolic reprogramming of energy allocation.

### 4.3. Molecular Mechanism of Synergistic Regulation of the Lipid Metabolism-Reproduction Axis in Ewes by Glycerol Complex

Previous studies demonstrated that glycerol absorbed via the rumen preferentially enters hepatic portal circulation, activating the AMPK/mTOR pathway to upregulate PEPCK (↑ 2.1-fold) and drive gluconeogenesis [29]. At the ovarian level, follicles possess an insulin-glucose-IGF-1 system modulated by short-term nutritional interventions, where glucose (Glu) serves as the primary metabolic fuel for fertility [30,31]. Berlinguer et al. in vitro follicular culture confirmed that high-glucose microenvironments (6.5 mmol/L) enhance granulosa cell mitochondrial membrane potential (Δψm ↑ 40%) and glucose uptake (↑ 55%) via GLUT4 translocation, improving oocyte maturation [32]. In this study, serum INS peaked at 22.7 ± 1.5 ng/mL on day 30 post-supplementation, potentially linked to ovarian IGF-1/PI3K-AKT pathway activation. Supplemental glycerol reshaped lipid metabolism balance, with Glu levels showing biphasic trends (peak: 4.2 ± 0.5 mmol/L at day 30), correlating with adipose tissue deposition-mobilization phases. These findings align with Sutton-McDowall’s report on glycogen-mediated oocyte quality enhancement; Rodent overfeeding models where sustained hyperglycemia stimulates LEP secretion via INS-LPL-FFA esterification, accelerating fat synthesis. Critically, glycerol supplementation induced phase-specific LEP fluctuations (decrease → increase), reducing leptin resistance and activating hypothalamic Kisspeptin/GnRH neurons to promote LH/FSH secretion [33]. Collectively, cyclic changes in circulating Glu, INS, and LEP establish a systemic metabolic environment conducive to hypothalamic–pituitary–ovarian axis reactivation during non-breeding seasons.

### 4.4. Mechanisms of Glycerol Complex Regulation of the Reproduction-Lipid Metabolism Axis in Ewes

Kisspeptin encoded by the kiss-1 gene drives pulsatile GnRH secretion through activation of the membrane receptor GPR54 in hypothalamic neurons, thereby promoting pituitary LH/FSH release (↑ 40% LH peak amplitude) [34,35,36]. This study demonstrated that glycerol supplementation significantly downregulated GnAQ expression (*p* < 0.01), alleviating its transcriptional repression on kiss-1 to activate the hypothalamic–pituitary–gonadal (HPG) axis (GnRH ↑, *p* < 0.05). These findings align with Zhou et al.’s report on high-energy diets promoting LHR expression [37]. In our study, LHR was significantly upregulated (*p* < 0.01), confirming the universality of energy-reproductive axis regulation. Synergistic actions of StAR (mediating mitochondrial cholesterol transport, *p* < 0.05) and CYP11A1 (catalyzing cholesterol side-chain cleavage, *p* < 0.01) enhanced pregnenolone synthesis efficiency, with serum P4 peaking on day 13 in synchrony with follicular luteinization. Concurrently, VEGF upregulation (*p* < 0.01) increased ovarian vascular density to support folliculogenesis [38]. The intervention promoted dual-pathway lipid mobilization: subcutaneous adipose HSL upregulation (*p* < 0.05) hydrolyzed triglycerides, while hepatic FFA oxidation provided cholesterol precursors for ovarian steroidogenesis (StAR ↑, CYP19A1 ↑). These metabolic reprogramming effects align with the “metabolism-reproductive coupling axis” theory in rodents [39], confirming glycerol’s tripartite regulatory network in non-seasonal estrus induction: Hepatic glucose homeostasis; Lipolysis-steroid synthesis coupling via cholesterol precursor flux; and LEP-Kisspeptin-GnRH neuroendocrine rhythm reset.

Since the original hypothalamic–ovarian tissues have been fully utilized for RNA extraction and histological analyses, the regulatory mechanisms of LHR, StAR, and CYP11A1 are currently based on mRNA-level data. Future studies should validate these mechanisms at the protein level using western blot or immunohistochemistry. In subsequent investigations, we will establish a primary cell model of the ovine hypothalamus/ovary to systematically examine the transcription-translation regulatory relationships of key genes.

### 4.5. Serum Metabolomic Profiling and Key Metabolite Resolution

Non-targeted metabolomics analysis based on LC-MS technology showed significant metabolic differences between the test groups (GLY, Tyr-GLY, B9-Tyr-GLY) and the control group (VIP > 1.5, FC > 1.2, *p* < 0.05). The differential metabolites were dominated by lipids and lipid-like molecules (68.3%), followed by organic acids and their derivatives (19.5%) and organic oxides (12.2%), which aligns with energy metabolism trends in ruminants.

Previous studies indicate that DL-carnitine mediates long-chain fatty acid transport to mitochondria via carnitine palmitoyltransferase I, promoting β-oxidation and enhancing energy metabolism efficiency [40,41,42,43]. Notably, Samir’s study demonstrated that DL-carnitine intervention elevated total antioxidant capacity, E2 and P4 in ewes [44], consistent with the significant DL-carnitine elevation (*p* < 0.001) and improved reproductive performance observed here. Glycerol phospholipid-like molecules were significantly enriched (*p* < 0.01) in the GLY group (vs. NC), suggesting glycerol regulates fat mobilization through phospholipid metabolism remodeling. In the Tyr-GLY and B9-Tyr-GLY groups, N-stearoyl taurine was significantly upregulated (*p* < 0.01) by Asp-Phe. Crucially, Asp-Phe (a prostaglandin analog) synchronizes estrous cycles via luteolysis, mirroring bovine estrus synchronization mechanisms reported by Yizengaw L [13]. This provides a theoretical basis for precision nutritional interventions in seasonal reproductive disorders.

## 5. Conclusions

This study confirmed that glycerol complexes restore the estrous cycle in ewes during the non-breeding season through a tripartite synergistic mechanism: (1) Metabolic–Energy Axis Regulation: The glycerol mixture activates Kisspeptin-GnRH neuronal activity in the hypothalamic arcuate nucleus, driving pulsatile LH/FSH secretion. Simultaneously, it enhances hepatic DL-carnitine synthesis efficiency via carnitine palmitoyltransferase I-mediated β-oxidation of long-chain fatty acids, providing energy support for follicular development. (2) Ovarian Function Optimization: DL-carnitine reduces mitochondrial oxidative stress, promotes granulosa cell proliferation, and stimulates steroid hormone synthesis (E2 ↑, P4 ↑). This approach provides a novel nutritional intervention strategy for seasonal reproductive regulation in ruminants.

## Figures and Tables

**Table 1:** Composition and nutritional components of the basic diet (of DM, %).

Item	Content, %	Nutrient Content ^2^	Content, %
Ingredients		(MJ/kg)	10.96
Alfalfa hay	40	DM	83.375
Alfalfa silage	35	CP	17.5
Corn Stover	5	Ash	8.63
Soybean Meal	7.9	EE	2.9
Corn	8.5	Ca	0.49
NaHCO_3_	1.5	P	0.41
premix ^1^	1	NDF	44.94
NaCl	0.8	ADF	35.59
Lys	0.3		
consider	100		

Note: ^1^ Each kilogram of premix contains 400,000 IU of vitamin A, 40,000 IU of vitamin D, 0.8 million IU of vitamin E, 3600 mg of iron, 4000 mg of zinc, 400 mg of copper, 2000 mg of manganese, 20 mg of Na, 20 mg of drill, and 40 mg of iodine. ^2^ Maintenance net energy values are calculated values, and the remaining nutrients are measured values. DM (Dry Matter); CP (Crude Protein); Ash (Crude Ash); EE (Ether Extract); Ca (Calcium); P (Phosphorus); NDF (Neutral Detergent Fiber); ADF (Acid Detergent Fiber).

**Table 2:** qRT-PCR primers (with 90–110% amplification efficiency).

Gene	Sequence (5′→3′)	Temperature/°C	Product Length/bp
GnAQ	F: GGACAGGAGAGAGTGGCAAG R: GGCCGTGAAGATGTTCTGAT	57	112
kiss-1	F: ATGAACGTGCTGCTTTCCT R: TCCGAGCTGCGAGCCTGTG	58	117
GPR54	F: CCTTCACCGCTCTGCTCTAC R: ACCGAGACCTGCTGGATGTA	58	88
GnRH	F: CTTAGGTTCTACTGGCTGAT R: TCCTGCTGACTTTCTGTG	58	112
CYP11A1	F: CGAGGGATCCTACCCACAGA R: GTTCTGGAGGGAGGTTGAGC	55	422
LHR	F: TGCGGCCTTTAATCGTTCCT R: ATACTACTGGGCCTGGGTGT	58	216
VEGF	F: GCACCGTCTTTTTGTCCCTC R: TCTTCCCAAAAGCAGGCCAA	58	103
StAR	F: GGCAGAGATGAGCCACACTT R: CCTGTGCCCCTTACCTTGAG	58	584
β-actin	F: AGAGCAAGAGAGGCATCC R: TCGTTGTAGAAGGTGTGGT	50~60	108
HSL	F: AGCACTACAAACGAACGA R: CTGAATGATCCGCTCAAACT	58	207
GAPDH	F: GTCGGAGTGAACGGATTTGG R: CATTGATGACGAGCTTCCCG	50~60	196

**Table 3:** Number of estrus ewes in each group and ovarian development of estrous ewes in the experimental group.

Group	Ewe in Heat	Non-Estrus Ewes	Estrus Rate	Number of Follicles/mm	Follicle Diameter/mm	Follicle Thickness/mm
Control	1	9	10%	-	-	-
GLY	3	7	30%	3 ± 1	1.8 ± 0.32	0.95 ± 0.25
GLY-Tyr	4	6	40%	3 ± 2	1.9 ± 0.18	0.85 ± 0.32
GLY-B9	3	7	30%	3 ± 1	2.2 ± 0.27	1.01 ± 0.43
GLY-Tyr-B9	5	5	50%	4 ± 2	2.4 ± 0.26	1.08 ± 0.53

**Table 4:** Distribution of differential metabolites in each comparison group (Partial).

Group	Metabolite Name	Formula	RT [min]	*m*/*z*	FC	*p*-Value	VIP	Trend
GLY.vs.NC	DL-Carnitine	C7 H15 N O3	1.330	162.112	2.655	0.000	2.215	↑
	4-Hydroxyisoleucine	C6 H13 N O3	1.832	148.097	1.456	0.002	1.915	↑
	Asp-Phe	C13 H16 N2 O5	4.852	281.113	1.751	0.003	2.131	↑
	PC O-17:0	C25 H52 N O7 P	9.249	510.354	1.439	0.012	1.259	↑
	LPC O-20:5	C28 H50 N O6 P	9.301	528.344	1.741	0.012	1.393	↑
	5-Hydroxyindole-2-carboxylic acid	C9 H7 N O3	2.834	178.050	0.663	0.016	1.654	↓
	Meperidine-d5	C15 H16 H5 N O2	6.694	253.195	0.700	0.021	1.309	↓
	gamma-Glutamyltyrosine	C14 H18 N2 O6	4.940	311.123	1.594	0.024	1.558	↑
	N-Methyllysine	C7 H16 N2 O2	1.243	161.128	2.454	0.027	1.820	↑
	Ornithine	C5 H12 N2 O2	1.179	133.097	1.530	0.042	1.532	↑
	D-Erythro-sphingosine 1-phosphate	C18 H38 N O5 P	8.438	380.255	0.807	0.044	1.333	↓
	morpholino(quinolin-6-yl)methanone	C14 H14 N2 O2	5.431	243.110	0.550	0.048	1.668	↓
	Ergosterol	C28 H44 O	7.929	397.346	2.299	0.048	1.662	↑
Tyr-GLY.vs.NC	Homoarginine	C7 H16 N4 O2	1.310	189.134	13.440	0.004	2.145	↑
	Ergosterol	C28 H44 O	7.929	397.346	2.975	0.004	2.029	↑
	3-(4-hydroxy-3-methoxyphenyl)propanoic acid	C10 H12 O4	5.587	219.063	0.643	0.004	2.192	↓
	Asp-Phe	C13 H16 N2 O5	4.852	281.113	1.522	0.005	1.428	↑
	N2-Acetyl-L-ornithine	C7 H14 N2 O3	1.415	175.108	0.627	0.007	1.631	↓
	DL-Carnitine	C7 H15 N O3	1.330	162.112	2.802	0.012	2.058	↑
	PC 17:0_17:0	C42 H84 N O8 P	11.127	762.595	0.490	0.027	1.680	↓
	Arachidonoyl amide	C20 H33 N O	8.730	304.263	2.575	0.027	1.658	↑
	L-Homocitrulline	C7 H15 N3 O3	1.414	190.118	3.535	0.027	1.795	↑
	DL-Arginine	C6 H14 N4 O2	1.280	175.119	0.772	0.029	1.408	↓
	Vitamin A	C20 H30 O	8.739	304.263	2.796	0.032	1.663	↑
	PC 36:2	C44 H84 N O8 P	9.883	808.581	0.447	0.034	2.037	↓
	N-Methyllysine	C7 H16 N2 O2	1.243	161.128	2.496	0.035	1.552	↑
	Creatinine	C4 H7 N3 O	1.383	114.066	1.452	0.040	1.408	↑
	D-Erythro-sphingosine 1-phosphate	C18 H38 N O5 P	8.438	380.255	0.788	0.043	1.326	↓
B9-GLYvs.NC	N-Methyllysine	C7 H16 N2 O2	1.243	161.128	2.547	1.349	0.010	↑
	L-Homocitrulline	C7 H15 N3 O3	1.414	190.118	3.367	1.751	0.011	↑
	Methyl palmitate	C17 H34 O2	6.740	288.289	2.226	1.155	0.021	↑
	N2-Acetyl-L-ornithine	C7 H14 N2 O3	1.415	175.108	0.647	−0.627	0.022	↓
	1-Methylhistidine	C7 H11 N3 O2	1.293	170.092	1.725	0.787	0.023	↑
	2-hydroxy-6-[(8Z,11Z)-pentadeca-8,11,14-trien-1-yl]benzoic acid	C22 H30 O3	8.180	343.222	1.738	0.798	0.024	↑
	Glycerophospho-N-palmitoyl ethanolamine	C21 H44 N O7 P	8.941	454.292	0.730	−0.454	0.033	↓
	LPC O-16:1	C24 H50 N O6 P	9.533	480.344	0.714	−0.486	0.042	↓
	Cystamine	C4 H12 N2 S2	1.245	153.052	0.766	−0.384	0.043	↓
	16-Heptadecyne-1,2,4-triol	C17 H32 O3	7.801	307.224	1.358	0.441	0.043	↑
	Deoxycytidine	C9 H13 N3 O4	1.409	250.079	0.593	−0.754	0.043	↓
	Cytosine	C4 H5 N3 O	1.880	112.051	0.577	−0.793	0.043	↓
	Lysopc 20:4	C28 H50 N O7 P	8.979	544.340	0.574	−0.800	0.045	↓
B9-Tyr-GLYvs.NC	DL-Carnitine	C7 H15 N O3	1.330	162.112	2.356	1.236	0.000	↑
	N-Formylkynurenine	C11 H12 N2 O4	4.888	237.087	2.744	1.456	0.000	↑
	PC O-38:5	C46 H84 N O7 P	11.299	794.604	0.501	−0.998	0.001	↓
	N-Stearoyl taurine	C20 H41 N O4 S	11.437	392.282	0.631	−0.664	0.002	↓
	Asp-Phe	C13 H16 N2 O5	4.852	281.113	1.507	0.591	0.003	↑
	Choline	C5 H13 N O	1.314	104.107	0.723	−0.468	0.005	↓
	gamma-Glutamyltyrosine	C14 H18 N2 O6	4.940	311.123	1.946	0.961	0.006	↑
	LPC 19:1-SN1	C27 H54 N O7 P	9.797	536.370	1.788	0.838	0.007	↑
	N-Acetylvaline	C7 H13 N O3	4.967	182.079	0.505	−0.985	0.009	↓
	Methyl palmitate	C17 H34 O2	6.740	288.289	2.126	1.088	0.012	↑
	Acetylcholine	C7 H15 N O2	1.395	146.117	1.413	0.498	0.014	↑
	Gly-Phe	C11 H14 N2 O3	5.234	223.107	1.956	0.968	0.014	↑
	N2-Acetyl-L-ornithine	C7 H14 N2 O3	1.415	175.108	0.615	−0.702	0.018	↓
	All trans-Retinal	C20 H28 O	6.453	285.221	1.674	0.743	0.038	↑
	Phenylacetylglycine	C10 H11 N O3	5.460	194.081	1.633	0.708	0.042	↑
	Ornithine	C5 H12 N2 O2	1.179	133.097	1.552	0.634	0.046	↑

**Table 5:** Enrichment results of KEGG Pathway for differential metabolites.

Group	MapID	MapTitle	*p*-Value	x	y	*n*	N
GLY.vs.NC	map04022	cGMP-PKG signaling pathway	0.07	1	1	6	92
	map04740	Olfactory transduction	0.07	1	1	6	92
	map04744	Phototransduction	0.07	1	1	6	92
	map00100	Steroid biosynthesis	0.13	1	2	6	92
	map01523	Antifolate resistance	0.13	1	2	6	92
	map04742	Taste transduction	0.13	1	2	6	92
	map04917	Prolactin signaling pathway	0.13	1	2	6	92
	map00760	Nicotinate and nicotinamide metabolism	0.19	1	3	6	92
	map01100	Metabolic pathways	0.33	6	69	6	92
	map04913	Ovarian steroidogenesis	0.34	1	6	6	92
	map04977	Vitamin digestion and absorption	0.34	1	6	6	92
	map00230	Purine metabolism	0.43	1	8	6	92
	map00140	Steroid hormone biosynthesis	0.58	1	12	6	92
Tyr-GLY.vs.NC	map00830	Retinol metabolism	0.02	2	2	13	92
	map04977	Vitamin digestion and absorption	0.20	2	6	13	92
	map00100	Steroid biosynthesis	0.26	1	2	13	92
	map00220	Arginine biosynthesis	0.37	1	3	13	92
	map00760	Nicotinate and nicotinamide metabolism	0.37	1	3	13	92
	map00330	Arginine and proline metabolism	0.46	1	4	13	92
	map00590	Arachidonic acid metabolism	0.46	1	4	13	92
	map04726	Serotonergic synapse	0.46	1	4	13	92
	map05215	Prostate cancer	0.46	1	4	13	92
	map01100	Metabolic pathways	0.51	11	69	13	92
	map00360	Phenylalanine metabolism	0.54	1	5	13	92
	map01210	2-Oxocarboxylic acid metabolism	0.54	1	5	13	92
	map05200	Pathways in cancer	0.54	1	5	13	92
	map00380	Tryptophan metabolism	0.63	2	10	13	92
	map00260	Glycine, serine and threonine metabolism	1.00	1	6	13	92
	map04913	Ovarian steroidogenesis	1.00	1	6	13	92
B9-GLY.vs.NC	map00240	Pyrimidine metabolism	0.00	4	5	8	92
	map01523	Antifolate resistance	0.17	1	2	8	92
	map00220	Arginine biosynthesis	0.24	1	3	8	92
	map01210	2-Oxocarboxylic acid metabolism	0.37	1	5	8	92
	map00260	Glycine, serine, and threonine metabolism	0.43	1	6	8	92
	map00340	Histidine metabolism	0.48	1	7	8	92
	map01230	Biosynthesis of amino acids	0.58	1	9	8	92
	map02010	ABC transporters	0.58	1	9	8	92
	map00380	Tryptophan metabolism	1.00	1	10	8	92
Tyr-B9-GLY.vs.NC	map01523	Antifolate resistance	0.01	2	2	11	92
	map04022	cGMP-PKG signaling pathway	0.12	1	1	11	92
	map04740	Olfactory transduction	0.12	1	1	11	92
	map04744	Phototransduction	0.12	1	1	11	92
	map05230	Central carbon metabolism in cancer	0.12	1	1	11	92
	map00410	beta-Alanine metabolism	0.23	1	2	11	92
	map04742	Taste transduction	0.23	1	2	11	92
	map04917	Prolactin signaling pathway	0.23	1	2	11	92
	map00230	Purine metabolism	0.24	2	8	11	92
	map04974	Protein digestion and absorption	0.24	2	8	11	92
	map01100	Metabolic pathways	0.28	10	69	11	92
	map01230	Biosynthesis of amino acids	0.29	2	9	11	92
	map00220	Arginine biosynthesis	0.32	1	3	11	92
	map00380	Tryptophan metabolism	0.34	2	10	11	92
	map00240	Pyrimidine metabolism	0.48	1	5	11	92
	map00360	Phenylalanine metabolism	0.48	1	5	11	92
	map00400	Phenylalanine, tyrosine and tryptophan biosynthesis	0.48	1	5	11	92
	map01210	2-Oxocarboxylic acid metabolism	0.48	1	5	11	92
	map04913	Ovarian steroidogenesis	0.54	1	6	11	92
	map00970	Aminoacyl-tRNA biosynthesis	1.00	1	8	11	92
	map04976	Bile secretion	1.00	1	8	11	92
	map00340	Histidine metabolism	1.00	1	7	11	92

## Data Availability

The data presented in this study are available upon request from the corresponding author. The data are not publicly available to preserve privacy.
